# Tailoring NiO-Based Nanostructures for the Electrochemical Valorization of Ethanol: Structure–Property Insights

**DOI:** 10.3390/nano16080496

**Published:** 2026-04-21

**Authors:** Ivan Blagojevic, Chiara Maccato, Marta De Zotti, Davide Barreca, Alberto Gasparotto, Raffaella Signorini, Gian Andrea Rizzi

**Affiliations:** 1 Department of Chemical Sciences, Padova University and National Interuniversity Consortium of Materials Science and Technology (INSTM), 35131 Padova, Italy; ivan.blagojevic@studenti.unipd.it (I.B.); marta.dezotti@unipd.it (M.D.Z.); alberto.gasparotto@unipd.it (A.G.); raffaella.signorini@unipd.it (R.S.); gianandrea.rizzi@unipd.it (G.A.R.); 2 Institute of Condensed Matter Chemistry and Technologies for Energy (CNR-ICMATE) and INSTM, Department of Chemical Sciences, Padova University, 35131 Padova, Italy; davide.barreca@cnr.it

**Keywords:** NiO, nanostructures, hydrothermal synthesis, water electrolysis, ethanol oxidation reaction

## Abstract

Water electrolysis has emerged as a strategically appealing route for the sustainable production of green hydrogen (H_2_) *via* the hydrogen evolution reaction (HER), though the sluggish kinetics of the oxygen evolution reaction (OER) remains a bottleneck hindering large-scale practical applications. In this regard, an attractive solution is offered by the integration of the ethanol oxidation reaction (EOR) into hybrid water-splitting systems, favorably reducing anodic overpotentials. Nonetheless, an open challenge is related to the fabrication of eco-friendly and economically viable catalysts free from noble metals, combining efficiency and stability. Herein, we explore nickel-oxide-based nanostructures grown onto porous Ni foam scaffolds by a scalable hydrothermal (HT) approach as EOR electrocatalysts. Material properties arising from modulation of the sole HT growth time are investigated by complementary structural, microscopic, and spectroscopic techniques. Electrochemical tests demonstrate good durability and very attractive EOR performances, mainly influenced by the morphology and the NiOOH surface content of the target systems. Overall, the present work advances an attractive route to transition-metal-based electrocatalysts for efficient alcohol-oxidation-assisted water electrolysis.

## 1. Introduction

Over the past decades, extensive research efforts have focused on the generation and exploitation of sustainable energy, in order to replace the widely used fossil fuels and promote societal development in a greener perspective [1,2,3,4]. In this context, molecular hydrogen (H_2_) has emerged as a green and renewable fuel of strategic interest [5,6], and water electrolysis stands as an appealing pathway for its production [7,8,9]. Conventional water splitting, consisting of the HER and the OER, requires a high overpotential due to kinetic limitations of OER, a slow four-electron transfer process [4,10,11]. Accordingly, several efforts have been undertaken to improve water oxidation and enhance its efficiency towards a possible large-scale exploitation [7]. To this aim, the substitution of OER with energy-saving and more favorable oxidation reactions opens the door to an innovative way for sustainable hydrogen production in a foreseeable future [12,13,14,15].

In this context, the ethanol oxidation reaction offers a dual opportunity, i.e., the reduction of energy requirements and the concomitant production of high-value-added chemicals [12,16,17]. In fact, ethanol (EtOH), easily produced by biomass fermentation, represents a promising renewable fuel thanks to its availability, carbon neutrality, easy storage, and high energy density (8.0 kWh/kg) [4,18,19,20,21,22]. Indeed, EOR proceeds at an appreciably lower overpotential than OER, and in alkaline media ethanol can be oxidized to acetate [17,23], a commodity far more valuable than molecular oxygen produced *via* OER [24,25,26]. Therefore, the development and engineering of high-performance EOR electrocatalysts are a key requirement for further scientific, technological, and economic advancements [4].

The current benchmark EOR catalysts are based on platinum, but their high cost and susceptibility to poisoning/deactivation preclude their extensive utilization [20,27]. These limitations have triggered a growing interest in the development of electrocatalysts minimizing, or avoiding, noble metal utilization, while optimizing the efficiency of transition-metal usage [3,20,28]. In this regard, nickel-based electrocatalysts and, in particular, Ni oxides and (oxy)-hydroxides stand out as valuable candidates for alkaline EOR thanks to their affordability, good conductivity, and favorable reaction kinetics [8,29]. Accordingly, several strategies have been explored to improve the performance of these materials, including the use of porous supports and the modulation of the system physicochemical characteristics through various synthesis/processing strategies [8,17,30]. Among the latter, hydrothermal growth processes represent advantageous alternatives thanks to their inherent versatility, empowered by numerous degrees of freedom enabling to modulate material morphology under a variety of operating conditions [31,32]. Accordingly, HT growth of NiO nanostructures on Ni foam (NF) substrates, combining a desirable open-pore structure with a high electrical conductivity [33], offers a tunable and relatively simple route toward the fabrication of customized materials for the target end-use. So far, different works have been focused on Ni-based EOR electrocatalysts [34,35,36,37,38,39,40], but only two studies have reported on the HT growth of NiO/Ni(OH)_2_ nanostructures on NFs [4,20], and in one case the target materials were engineered through Ru introduction [20].

Within the above literature context, the present study proposes a simple HT method for the fabrication and mastering of noble metal-free NiO-based electrocatalysts on NF substrates. It is worth highlighting that, at variance with different papers on Ni-based EOR electrocatalysts, the preparative strategy proposed herein is quite straightforward, avoiding complex reaction mixtures aimed at fabricating, for instance, doped or composite systems. Importantly, it allows the direct, in situ growth of the catalyst material over the Ni foam substrate. In this way, drawbacks commonly encountered during the immobilization of pre-synthesized powdered catalysts (e.g., need for binders, poor uniformity and limited mechanical adhesion of the deposit) can be overcome, and the active phase can benefit from enhanced contact and dispersion with/within the substrate.

A comprehensive compositional, structural, and morphological characterization underscored the possibility of tailoring material properties through variations of the sole growth time (between 15 and 60 min). Electrochemical tests revealed that the best-performing catalyst yielded a Tafel slope as low as 48 mV/dec and a current density of 150 mA/cm^2^ at 1.6 V vs. the reversible hydrogen electrode (RHE). These outcomes, accompanied by a good material durability, are well-positioned in the current panorama of actual Ni-based electrocatalysts. The analysis of reaction products enabled us to shed light on the oxidation process occurring under the adopted conditions. Taken together, the present results highlight the potential of the developed electrocatalysts for the production of clean energy *via* EtOH valorization, coupled with the generation of value-added chemicals.

## 2. Materials and Methods

### 2.1. Material Preparation 

**Figure 1:**
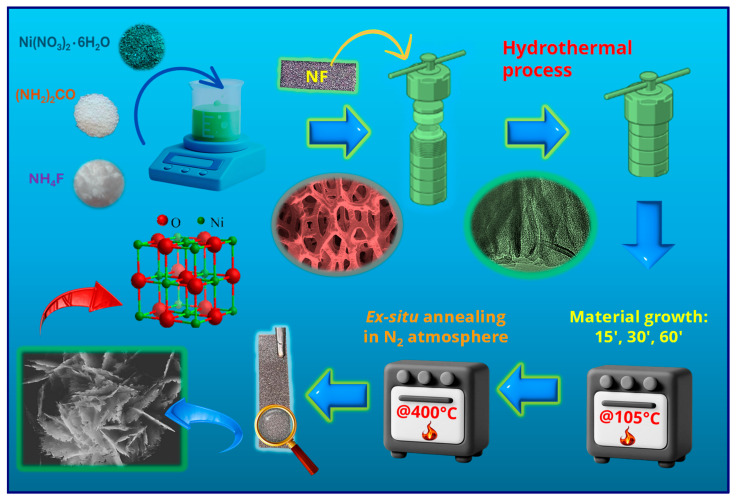
Sketch of the fabrication procedure adopted for the growth of the present electrocatalysts on NF substrates.

NiO nanostructures were grown following a procedure described by Chen et al. [41] for the fabrication of porous NiO films on conducting glasses. In a typical synthesis, the following reactants were dissolved in 15.0 mL of deionized water: 1.25 g/L of Ni(NO_3_)_2_●6H_2_O, 0.32 g/L of NH_4_F, and 1.30 g/L of NH_2_CONH_2_ (Sigma-Aldrich, St. Louis, MI, USA) The obtained solution was transferred into a Teflon-lined vessel, and pre-cleaned [33] Ni foam substrates (Recemat BV, Dodewaard, The Netherlands; dimensions: 4 cm × 1 cm) were immersed in the reaction medium. The liner was sealed in a stainless-steel autoclave and heated at 105 °C for different growth times (15, 30, 60 min; heating rate = 3 °C × min^−1^). After cooling to room temperature, the substrate was removed, rinsed with deionized water, and dried in air. The obtained systems were finally subjected to an ex situ thermal treatment at 400 °C for 2 h under a N_2_ atmosphere (heating rate = 5 °C × min^−1^). The complete preparation procedure is sketched in Figure 1.

### 2.2. Material Characterization

X-ray diffraction (XRD) patterns were recorded using a Bruker (Karlsruhe, Germany) AXS D8 Advance Plus instrument equipped with a Göbel mirror and a CuKα X-ray source (λ = 1.54051 Å), powered at 40 kV and 40 mA, operating in glancing incidence mode (θ_i_ = 1.0°). The utilized Raman set-up comprises an Ar^+^ laser (Spectra Physics, λ = 514 nm). The laser is integrated with an Olympus BX 40 microscope equipped with 4×, 10×, 20×, 50×, and 100× magnification objectives (Olympus SLMPL series, Tokyo, Japan). The apparatus incorporates edge and notch filters, allowing for the selection of Raman Stokes transitions and removing the elastic scattering contribution from the resulting spectra. The scattered light travels through a Triax-320 spectrograph (ISA Instruments, Jobin Yvon, Rome, Italy) and is then detected by a liquid nitrogen-cooled charge-coupled device (CCD; Horiba Scientific Symphony II FIOE model, Rome, Italy), ensuring low noise and high sensitivity in the spectral measurements. X-ray photoelectron spectroscopy (XPS), ultraviolet photoelectron spectroscopy (UPS), and reflection electron energy loss spectroscopy (REELS) analyses were performed using a ThermoFisher (Waltham, MA, USA) ESCALAB QXi apparatus, utilizing a standard Al Kα X-ray source (hν = 1486.6 eV) operated at 200 W. Survey spectra were recorded at 100 eV pass energy (PE), 1.0 eV/step, and 0.05 s/step. High-resolution spectra were collected at 50 eV PE, 0.05 eV/step, and 0.1 s/step. Binding energies (BEs; uncertainty = ±0.1 eV) were corrected for charging phenomena by positioning the adventitious component of the C1s signal (Figure S3) at 284.8 eV [42]. Peak fitting was carried out using XPSPeak software, version 4.1 [43] and applying a Shirley-type background subtraction. Atomic percentage (at. %) data were obtained by peak integration, using ThermoFisher sensitivity factors. UPS analysis was performed using a UV He(I) photon source (hν = 21.22 eV), with the following settings: 2.0 eV PE; 0.01 eV/step; 0.05 s/step. Work function measurements were performed on un-sputtered specimens [8] in *bias-on* mode (−3 V) [44]. REELS spectra were recorded with a primary beam energy of 1.0 keV (PE = 3.0 eV; 0.05 eV/step; 0.01 s/step) and subjected to background subtraction according to the Tougaard model [45]. Field emission scanning electron microscopy (FE-SEM) analyses were carried out using a Zeiss (Oberkochen, Germany) SUPRA 40 VP apparatus, with primary beam acceleration voltages between 10 and 20 kV. The mean nanoaggregate size and deposit thickness values were calculated using ImageJ^®^ software (version 1.54s) [46]. Selected area electron diffraction (SAED) and energy dispersive X-ray spectroscopy (EDXS) analyses were performed on a ThermoFisher Scientific Titan probe-corrected apparatus, equipped with a Super-X windowless EDXS detector system operated at 300 kV. High resolution transmission electron microscopy (HRTEM) images were acquired at an acceleration voltage of 300 kV using a FEI (Hillsboro, OR, USA) Titan 80–300 double-corrected microscope. TEM images were acquired on a scratched sample portion. ^13^C nuclear magnetic resonance (NMR) experiments were performed on a Bruker (Billerica, MA, USA) 400 Avance III HD instrument, operating at 100.6 MHz for C, equipped with a BBI-z grad probehead, at 298 K. Spectra were processed using Bruker TopSpin 3.5 (Bruker BioSpin GmbH, Rheinstetten, Germany) and analyzed with MestReNova 6.0.2 (MESTRELAB RESEARCH, S.L.U., Santiago de Compostela, Spain). A H_2_O/D_2_O 9:1 mixture was used as solvent. Chemical shifts are reported in parts per million (ppm).

### 2.3. Electrochemical Tests

Electrochemical tests were carried out with an Autolab PGSTAT204 workstation (Utrecht, The Netherlands), using a Hg/HgO (MMO) electrode, a Pt coil, and NF-supported samples as reference (RE), counter (CE), and working electrodes (WE), respectively. To ensure a reliable electrical connection, the uncovered area of each NF substrate was trimmed (1 mm) to expose fresh metallic Ni, and a Ni-plated Cu wire was soldered to the foam using tin solder flux. The soldered junction was subsequently encapsulated within a two-component epoxy resin, that was allowed to rest overnight prior to electrochemical testing. Potential values vs. MMO (E_MMO_) were converted into the RHE scale using the following equation [21,28]:

E (V) = E_WE_ (V) + E_MMO_ (V) + 0.0592 × pH (1)

where E_WE_ indicates the working electrode potential. Linear sweep voltammetry (LSV) curves were initially recorded in 1.0 M KOH solution and then in 1.0 M KOH + 1.3 M EtOH solution (scan rate = 1 mV/s). Cyclic voltammetry (CV) measurements were carried out using a scan rate of 10 mV/s. Chronoamperometric (CA) analyses were performed at a fixed bias value of 1.60 V vs. RHE. Current density (j) values were obtained by normalizing the registered currents to the geometric electrode area (3 cm^2^). Tafel slopes were determined by the plots of potential vs. log(current density) [21,28].

## 3. Results and Discussion

### 3.1. Chemico-Physical Characterization

**Figure 2:**
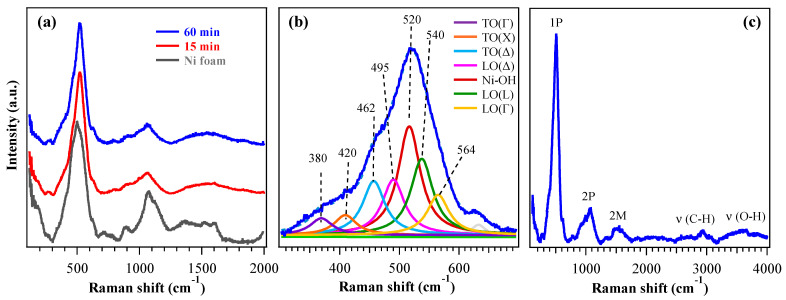
(**a**) Raman spectra of specimens obtained with growth times of 15 min and 60 min, from 200 to 2000 cm^−1^. The bare NF spectrum is also plotted for comparison. (**b**) Fitting of 1P peak for the 60 min sample, with corresponding band assignments. (**c**) Raman spectrum of the 60 min specimen up to 4000 cm^−1^.

The target systems were preliminarily analyzed by XRD (Figure S1). Since the recorded patterns exhibited only peaks pertaining to the NF support, Raman spectroscopy analyses were carried out to gain a deeper structural insight. Figure 2a shows typical Raman spectra acquired from 200 up to 2000 cm^−1^, revealing characteristic vibrational features of cubic nickel(II) oxide. Besides the two-magnon (2M) band at ≈1500 cm^−1^, characteristic of the antiferromagnetic order in bulk nickel(II) oxide [47], Raman spectra showed a very intense peak around 500 cm^−1^ (Figure 2b), associated with first-order phonon modes (1P) [48]. In bulk crystals with the rocksalt structure as NiO, transverse (TO) and longitudinal (LO) optical modes are Raman-inactive and become visible only in the presence of defects or distortions, although with lower intensity [49,50]. In nanostructured materials such as the present ones, however, both variations in the Ni:O stoichiometry and structural disorder relax Raman selection rules, enabling strong activation of 1P modes. According to the literature, TO and LO modes fall in the 400–600 cm^−1^ range and are associated with magnetostriction-induced vibrations (TO) and non-stoichiometric Ni–O stretching (LO). In addition, the LO/TO Raman intensity ratio has been reported to increase as the NiO grain size decreases [50]. Figure 2b shows the multiple contributions to the 1P band which include, according to Wang et al. [50], the distinction of TO (350–460 cm^−1^) and LO (495–580 cm^−1^) modes at different positions in the Brillouin zone, i.e., Γ (center), X (edge along the [100] direction), Δ (intermediate between the two previous ones) and L (edge along the [111] direction). Nonetheless, at variance with a previous work [50], a further component at 520 cm^−1^ was observed and attributed to Ni–OH vibrational modes [51,52,53] (in agreement with XPS, see data below). A direct comparison of the peak at 500 cm^−1^ for the 60 min sample and the substrate (Figure S2a) highlights the variation in the signal, revealing different contributions from the underlying Raman modes, particularly the one due to the Ni–OH vibrations. The corresponding curve fit for NF (Figure S2b) shows components similar to those of Figure 2b, but with different intensities. In particular, the peak at 520 cm^−1^ (due to Ni–OH vibrational modes) has a much higher intensity in the sample, whereas the peaks at ≈460 and 495 cm^−1^ [TO(Δ) and LO(Δ)] have a greater intensity in NF. The signal observed at higher frequencies in the 800–1200 cm^−1^ range (Figure 2a) corresponded to the second-order phonon (2P) region. It could be attributed to TO+LO at 900 cm^−1^, together with 2LO modes at 995 and 1060 cm^−1^ [48]. In bulk NiO these bands are well-defined, whereas in nanosystems such as the present ones they appear larger, less intense and displaced due to structural disorder and the overlap of multiple phonon contributions [49]. The full Raman spectrum (Figure 2c) also displays C–H (≈2930 cm^−1^) and O–H (≈3500 cm^−1^) stretching bands, suggesting the occurrence of some organic contamination and surface hydroxylation. These outcomes are consistent with XPS ones and, in particular, the O–H band can be traced back to the presence of Ni(OH)_2_ and NiOOH, providing further support for the assignment of the 1P contribution at 520 cm^−1^.

**Figure 3:**
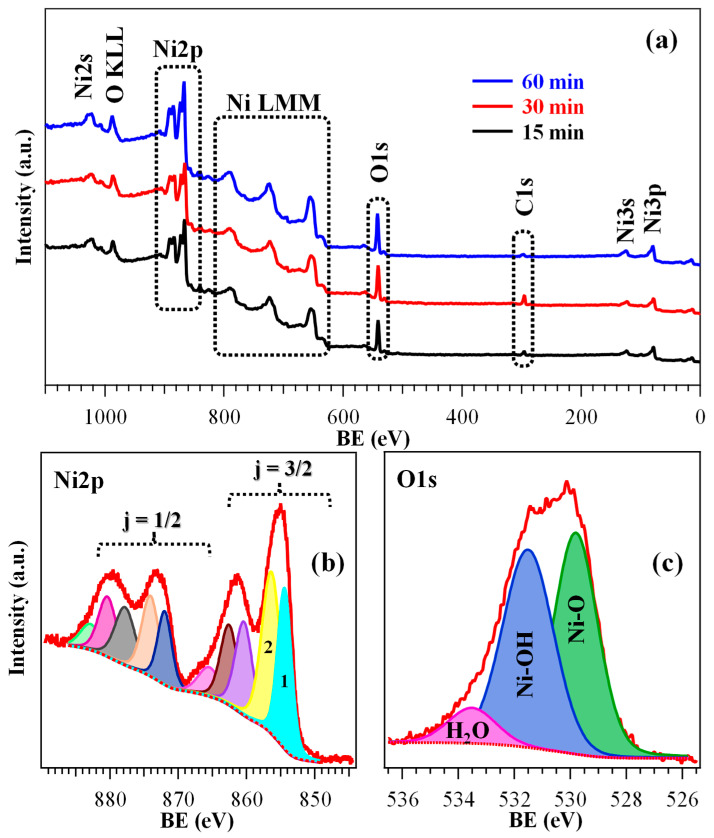
(**a**) Wide-scan XPS spectra of specimens fabricated using different HT growth times. High-resolution Ni2p (**b**) and O1s (**c**) signals for the 30 min sample, along with the corresponding contributing components. A detailed band assignment is proposed in Tables S2 and S3.

To gain insight into the surface composition and element chemical state, XPS analysis was performed on all the specimens. Wide-scan spectra (Figure 3a) showed the expected signals for Ni, O, and C (see also Figures S3–S5 and Tables S1–S3). The relatively low carbon content (<10 at. %) highlighted a good purity of the obtained materials.

As a matter of fact, Ni2p photopeak analysis (Figure 3b and Figure S4; Table S2) required particular attention, being overcomplicated by the presence of multi-electron interactions resulting in the appearance of a peculiar satellite structure [8,44,48,54]. As concerns the j = 3/2 spin–orbit contribution, the intensity ratio between the two low BE components (labeled as **1** and **2** in Figure 3b, and associated respectively with Ni^2+^ and Ni^3+^) can act as a qualitative indicator of the relative Ni^2+^/Ni^3+^ content, the latter oxidation state being associated with O defects [20,30]. Specifically, the obtained data (see Tables S2 and S3) suggested the formation of a hydroxylated surface, resulting from the coexistence of Ni(OH)_2_ and NiOOH [15,30]. The latter, present in a higher amount for the 30 min sample, can indeed exert a promotional effect on the target electrocatalytic process [7,14,55]. The higher hydroxylation level for the 30 min specimen was confirmed even by O1s signal analysis (Figure 3c and Figure S5; Table S3). In fact, the O/Ni at. % ratios were estimated to be 1.05, 1.35, and 1.00 for the 15 min, 30 min, and 60 min specimens, respectively. In this regard, it is important to highlight that hydroxylation and NiOOH formation in the present samples were confined to the system surface, since both Raman spectroscopy and electron diffraction, sensitive even to the inner material regions (see above and below, respectively), confirmed the bulk presence of NiO.

**Figure 4:**
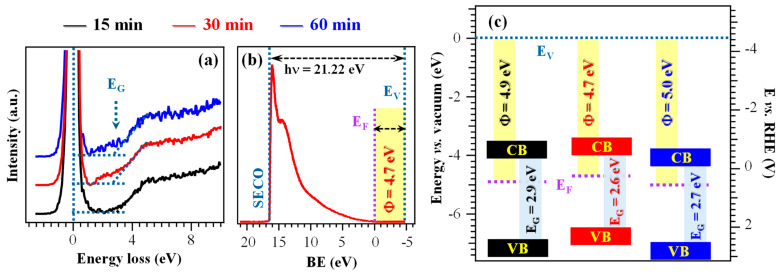
(**a**) REELS spectra of the target samples. (**b**) UPS valence band for the sample fabricated using a growth time of 30 min. (**c**) Band scheme for the investigated specimens. E_G_ = band gap; SECO = secondary electron cut-off; E_F_ = Fermi energy; E_V_ = vacuum level energy; Φ = work function; CB = conduction band; VB = valence band.

Information on material electronic structure was obtained from combined REELS and UPS analyses (Figure 4a,b and Table S4). The REELS spectra (Figure 4a), dominated by the intense peak at zero energy loss resulting from elastic scattering, yielded electronic band gap (E_G_) values between 2.6 and 2.9 eV (Figure 4c and Table S4), consistent with literature values for Ni(OH)_2_. In contrast, pristine NiO presents a larger band gap, around 3.5 eV [8,44]. These data, in line with XPS results, further supported the presence of surface hydroxylation [15,44,56]. The higher hydroxylation level of the 30 min specimen is consistent with its slightly narrower E_G_ (Table S4), suggesting an improved electronic conductivity in comparison to the other electrocatalysts [15]. These conclusions were further corroborated by the calculated work functions, that were relatively similar (Φ = 4.7÷5.0 eV, see Figure 4b,c and Table S4) [30,57], and by the separation of valence band edges from Fermi levels. The latter values were in agreement with an *n*-type character of the outermost layers, at variance with the case of pure NiO films and nanosystems [8]. The resulting band schemes (Figure 4c) exhibited only modest variations with growth time, implying that the differences in functional performance among the studied systems are primarily driven by other factors than the electronic structure itself.

As a whole, XPS and UPS data point out the co-presence of Ni(OH)_2_ and NiOOH and the surface hydroxylation of electrocatalysts. The main difference in the system electronic properties is the lower gap value of the 30 min sample, one of the issues responsible for its better functional performances (see below). 

**Figure 5:**
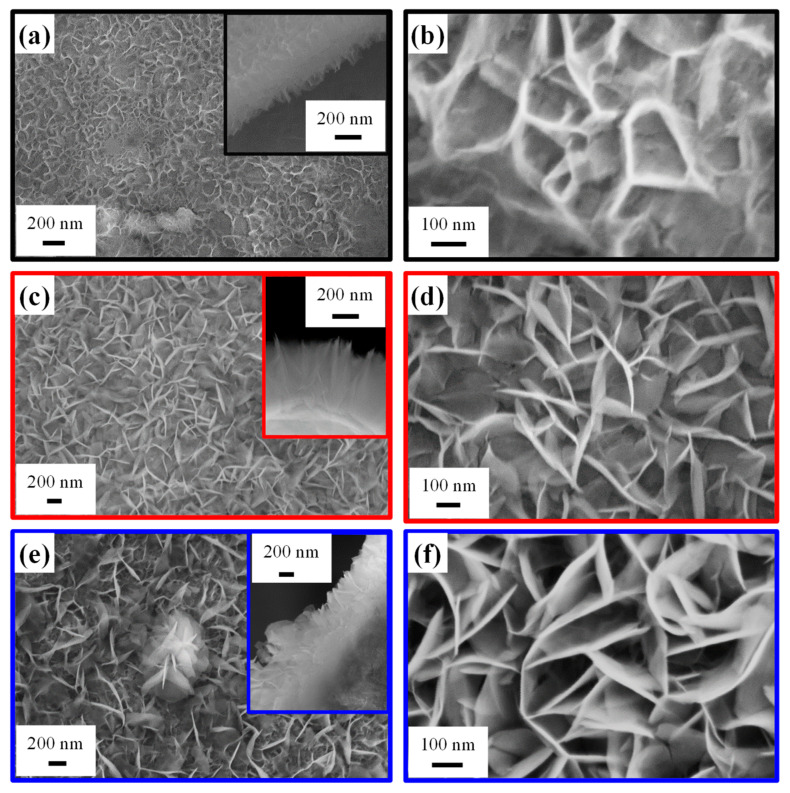
Representative FE-SEM images for samples obtained using an HT growth time of 15 min (**a**,**b**), 30 min (**c**,**d**), and 60 min (**e**,**f**). Cross-sectional micrographs are reported as insets in panels (**a**,**c**,**e**).

Efforts were subsequently devoted to the analysis of the system morphology. FE-SEM micrographs (Figure 5) revealed sheet-like structures grown nearly perpendicular to the underlying NF substrate and interconnected with each other, forming a high-area 3D network. This architecture provides a high surface-to-volume ratio, favorable for electrocatalytic applications, and a high density of active sites, enhancing mass transport and reaction kinetics [4,8,20]. Interestingly, the system morphology, qualitatively similar to previously reported Ru-decorated NiO and Ni(OH)_2_ catalysts [4,20], was directly dependent on the adopted growth time. In fact, based on a statistical FE-SEM image analysis, the nanosheet thickness (length) was estimated to be 6 nm (60 nm), 11 nm (120 nm), and 15 nm (280 nm) for the 15 min, 30 min, and 60 min samples, respectively. The mean deposit thickness was 350 nm for a process duration of 15 and 30 min, whereas it underwent an increase to 620 nm for the 60 min specimen. A more detailed image inspection revealed an island growth mode for the 15 min specimen, with the NF exhibiting a variable extent of surface coverage across different regions. Conversely, growth durations of 30 and 60 min resulted in a regular coverage of the NF surface by interwoven nanosheets forming a continuous network. For the 60 min specimen, some flower-like structures (one of which is visible in the center of Figure 5e) were also observed atop the underlying nanosheet network.

Overall, morphological data presented so far suggest that a deposition time of 30 min enables a sufficiently high active surface exposed to the outer environment, thus producing an increased hydroxylation level and a higher Ni^3+^ content (see above) in comparison to the 15 min sample. In a different way, for a growth time of 60 min, the larger material amount leads to the formation of flower-like aggregates, which, in turn, are responsible for a reduction of the overall active area. In addition, the deposit thickness for the 60 min electrocatalyst is too high to ensure a good conductivity and, therefore, an efficient system polarization. Consequently, HT growth for 30 min corresponds to an optimal compromise in terms of material features, with a direct impact on EOR performances (see below).

**Figure 6:**
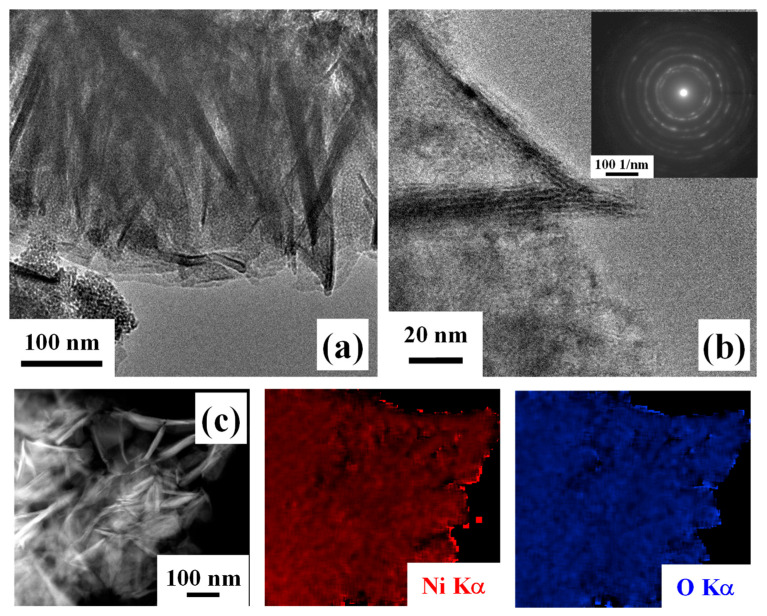
Low- (**a**) and high-resolution (**b**) TEM images for a specimen obtained using an HT growth time of 30 min. The SAED pattern is shown as inset in (**b**). (**c**) EDXS Ni and O elemental maps recorded on the corresponding electron image.

Further insights into material nano-organization were attained by TEM analyses. Low-magnification imaging (Figure 6a), in agreement with FE-SEM outcomes, highlighted the occurrence of thin and interconnected nanosheets. HRTEM investigation (Figure 6b) revealed well-defined lattice fringes, indicative of the system crystallinity. In fact, the corresponding SAED pattern (Figure 6b, inset) was dominated by well-defined concentric rings that could be assigned to polycrystalline cubic NiO, in line with Raman spectroscopy results [58]. In addition, EDXS analyses (Figure 6c) confirmed the homogeneous distribution of both nickel and oxygen, supporting an even NiO formation. Altogether, these results, combined with XPS and UPS outcomes, indicate that the target systems are characterized by the coexistence of NiO and of surface Ni(OH)_2_ and NiOOH species.

### 3.2. Functional Tests

**Figure 7:**
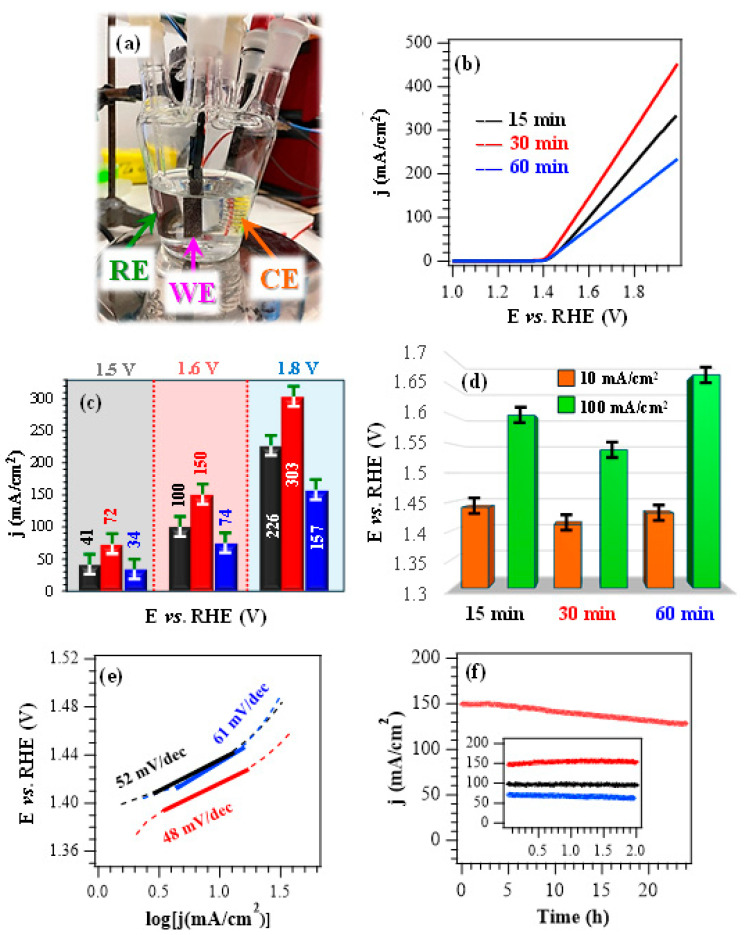
(**a**) Photograph of the cell used for electrochemical functional tests. (**b**) LSV scans for the target specimens registered in a 1.0 M KOH + 1.3 M EtOH solution. (**c**) Current density at different potential values for the same electrocatalysts. (**d**) Potentials required to reach current densities of 10 and 100 mA/cm^2^, extracted from the curves plotted in (**b**). (**e**) Corresponding Tafel plots. (**f**) CA tests carried out at a fixed potential of 1.60 V vs. RHE. Color codes in (**c**,**e**,**f**) as in panel (**b**).

After chemico-physical characterization, efforts were aimed at assessing material performances in the ethanol oxidation reaction. If not otherwise stated, electrochemical experiments (Figure 7a) were conducted in alkaline media at *pH* = 14.0, with an EtOH concentration of 1.3 M, consistent with previous studies [28]. Preliminary tests performed in the absence of ethanol evidenced that, upon increasing the *pH* from 13.0 to 14.0, LSV curves showed the appearance of a peak located between 1.4 V and 1.5 V vs. RHE, corresponding to the increased formation of NiOOH active species (Figure S6a,b) [8]. A comparison of LSV curves registered in KOH during control experiments (Figure S6b) with those obtained in KOH + EtOH (Figure 7b; see also Figure 7c) revealed that EtOH introduction in the working solution resulted in a marked current density enhancement, thus highlighting the EOR contribution to the registered j values. The bias values necessary to reach current densities of 10 mA/cm^2^ and 100 mA/cm^2^ (Figure 7d) are considerably lower than those for the OER (see the caption for Figure S6b), thus underscoring that EOR proceeds at substantially lower bias in the same operating conditions. These data highlight the potential of the present catalysts in hybrid electrolysis systems coupling hydrogen evolution with added-value oxidation reactions [20].

In the presence of ethanol, LSV curves (Figure 7b) do not feature the typical oxidation peak associated with NiOOH formation. This phenomenon can be attributed to the high electrocatalyst reactivity towards EOR, considering that the formed NiOOH immediately participates to EOR, is reduced to Ni(OH)_2_, and is periodically regenerated during testing. According to previous works [59,60], EOR processes on Ni(OH)_2_ surface involve both the electrochemical oxidation of Ni^2+^ centres into Ni^3+^ and the subsequent dehydrogenation of Ni^2+^–OH moieties. In addition, when the dehydrogenation of ethanol C–H bonds takes place, the concomitant reduction of Ni^3+^ back to Ni^2+^ occurs. These multistep mechanisms can explain the Ni redox cycling between Ni^2+^ and Ni^3+^ species.

Nevertheless, upon injection of small EtOH amounts, two distinct anodic peaks emerge (Figure S7), which can be considered the fingerprint of two subsequent oxidative steps [29]. In this scenario, the sample delivering the highest current densities throughout the investigated potential range is the one deposited for 30 min (Figure 7b,c). The better performances of this system in comparison to the other fabricated electrocatalysts are also highlighted by the calculation of Tafel slopes (Figure 7e and Table S5), since the lowest value of 48 mV/dec corresponded to the 30 min specimen. This result reflected an accelerated reaction kinetics, consistent with an enhanced conductivity (see the obtained band gap values, above and in Table S4) in comparison to the 15 min and 60 min electrocatalysts [4,20]. A comparison of the key performance indicators with those of various Ni oxide, hydroxide, and sulfide-based EOR electrocatalysts with different compositions (Table S5) highlights that the present systems are well-positioned in terms of delivered currents, working potentials, and Tafel slope values. This result underscores their promise for an eventual real-world implementation in alcohol-assisted water electrolysis.

The data presented so far indicate that, under the adopted conditions, the EOR performance trend is 60 min < 15 min < 30 min (see Figure 7). The superior activity of the 30 min specimen can be explained based on the concurrence of compositional and morphological characteristics. In fact, the 30 min electrocatalyst features a higher content of Ni^3+^ active species (see above), exerting a favorable promotional effect on the EOR process. The latter is also promoted by the slightly lower gap values in comparison to the other systems, accounting for an improved electronic conductivity. In addition, the 30 min electrocatalyst presents an even morphology featuring evenly distributed and interwoven nanosheets, expected to contribute to an enhanced charge carrier transport. On the other hand, the appreciably higher deposit thickness and the outermost occurrence of nanoflowers in the case of the 60 min specimen result in worse EOR performances, thus accounting for the observed activity sequence.

The operational durability of the developed systems, an important prerequisite for their practical end-use [4], was investigated by CA tests (Figure 7f). For an overall duration of 2 h, all electrocatalysts presented a very limited current density loss, in particular the 30 min one, in line with its higher electrochemical performances. Material stability was also corroborated by the negligible variations displayed by LSV traces upon repeated testing (Figure S8). In a different way, CA analyses extended to 24 h revealed a current density decrease over time. This effect was traced back to ethanol consumption and, in fact, a periodical electrolyte renewal was performed in previous studies to minimize the impact of this phenomenon [4,7,20]. *Post operando* FE-SEM analyses (Figure S9) enabled us to rule out the occurrence of remarkable morphological alterations, while XPS investigation evidenced that the interaction with KOH led to an increased formation of both Ni(OH)_2_ and NiOOH [4,8]. This conclusion was corroborated by a comparison of Ni2p and O1s peaks (Figure S10) with those of the pristine specimens (Figure 3b,c, Figures S4 and S5) and, in particular, by the increase of the Ni-OH contribution (at BE ≈ 531.5 eV) to the overall O signal.

**Figure 8:**
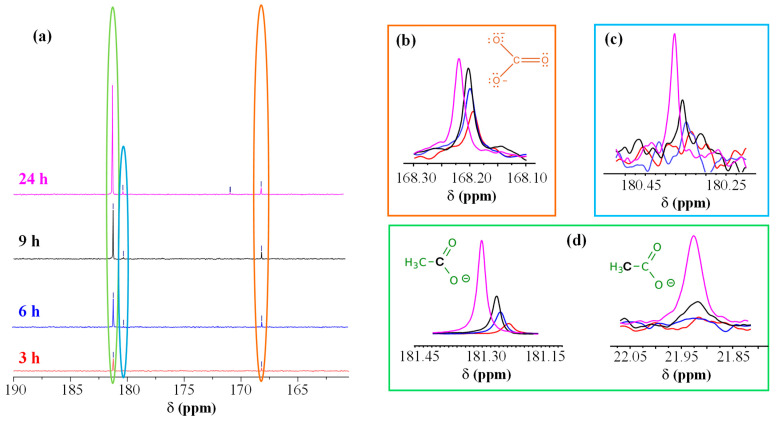
(**a**) Carbonyl region of the ^13^C NMR spectra acquired after reaction for 3 h (red), 6 h (blue), 9 h (black), and 24 h (magenta) with the electrocatalyst synthesized using an HT growth time of 30 min. Evolution of carbonate (**b**), glycolate (**c**), and acetate carboxyl peaks (**d**) as a function of reaction time [color codes as in panel (**a**)]. For acetate (**d**), even the signal of the methyl C atom is plotted as a function of reaction time.

To elucidate the process mechanism, the electrolyte solutions before CA and at different reaction times (3, 6, 9, and 24 h of CA testing) were analyzed by NMR (Figure 8 and Figures S11–S14). Figure 8a–d show respectively: (a) the carbonyl region of ^13^C NMR spectra as a function of CA reaction time; (b) the development of carbonate peak as a function of CA duration; (c,d) the time evolution of peaks associated with an aldehyde (c, described below) and acetate (d), which provided insights on EtOH oxidative fate. After 3 h (Figure S12), two ^13^C NMR signals were detected [at ≈23 ppm (a quartet) and 181 ppm] beside ethanol ones. These additional signals were assigned to acetic acid/acetate (–CH_3_ and C=O, respectively), a commodity of significant importance for the chemical industry [17]. The intensity of acetate resonances increased with reaction time, indicating a continuous EtOH oxidation on the catalyst [20]. The signal at ≈168 ppm, attributed to carbonate, was consistent with the oxidation under basic conditions, involving the breaking of the C–C bond leading to the formation of carbon dioxide, which, in alkaline media, is converted into carbonate according to the reaction: CO_2_ + 2OH^−^ ⇌ CO_3_^2−^ + H_2_O. Figure S13 shows the spectrum of the solution collected after 6 h of CA, where two further signals at ≈46 and 180 ppm emerged. This pattern initially suggested the presence of acetaldehyde, a key intermediate in EtOH oxidation, though the peak located at 180 ppm (Figure 8a,c) fell at quite lower ppm than typical aldehydic carbonyls (above 190 ppm).

A similar shift might depend on solvent effects, *pH*, or hydration phenomena. Under aqueous/oxidative conditions, acetaldehyde is often partially hydrated (gem-diol form), which can also induce the carbonyl signal shift. In order to confirm the assignment, we carried out NMR experiments without ^1^H decoupling to evaluate ^13^C multiplicity. The peak at ≈180 ppm appeared as a singlet, whereas the one at ≈46 ppm as a triplet (Figure S13). Since acetaldehyde would typically yield a doublet at ≈190 ppm (CHO) and a quartet at ≈30–45 ppm (CH_3_), we attributed these peaks to glycolate (HO–CH_2_–COO^−^), also taking into account that, under strongly alkaline oxidation conditions, EtOH can indeed form such C_2_ oxygenated species [17]. The glycolate methylene signal typically appears around 60 ppm, but in basic solutions it can shift towards 45–55 ppm. After 24 h (Figure S14), additional weak ^13^C peaks emerged, in particular one at ≈171 ppm that appeared as a doublet from NMR experiments without ^1^H decoupling. A similar result suggested the presence of a small amount of glyoxylate (O=CH–COO^−^), generated by a further glycolate oxidation. Even in this case, the aldehyde signal may shift at lower ppm than the typical 190–200 ppm, especially when hydrated. Indeed, in the present reaction environment, glyoxylate likely exists predominantly as its hydrated gem-diol form (di-hydroxy-acetate) rather than the free aldehyde.

**Figure 9:**
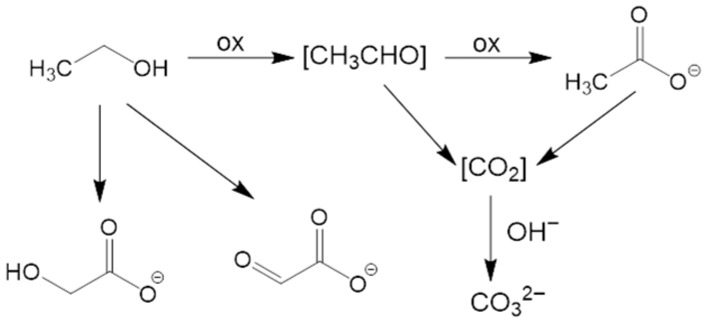
Schematic diagram for the possible pathways of ethanol oxidation.

Based on ^13^C NMR investigation, which identified acetate as the main product, along with carbonate, and glycolate/glyoxylate as minor products, we devised a possible pathway for EtOH oxidation. The mechanism, sketched in Figure 9, involves both a direct oxidation to acetate and C–C bond cleavage to yield carbonate, and a secondary pathway leading to C2 hydroxylated species. Ethanol is first oxidized to an aldehyde intermediate, which, in turn, is rapidly further oxidized to acetate, the primary product identified by NMR after 3 h of reaction. Simultaneously, a fraction of ethanol (or its intermediates) undergoes a complete oxidation involving C–C bond cleavage. This leads to the formation of CO_2_, which reacts with the alkaline electrolyte to produce carbonates (CO_3_^2−^), as confirmed by the NMR signal at 168 ppm:

CH_3_CH_2_OH + NiOOH → [CH_3_CHO] → CH_3_COO^−^ + Ni(OH)_2_ (2)

CH_3_CH_2_OH + 16 OH^−^ → 2 CO_3_^2−^ + 11 H_2_O + 12e^−^ (complete oxidation) (3)

As the reaction time increases (from 6 h to 24 h), NMR spectra reveal additional signals corresponding to glycolate (HO–CH_2_–COO^−^) and, in smaller amounts, glyoxylate (O=CH–COO^−^). These evidences indicate a secondary pathway where the C2 chain is progressively hydroxylated/oxidized without C–C bond cleavage.

## 4. Conclusions

In summary, this work advances the fabrication and customization of NiO-based nanostructures supported on Ni foams as electrocatalysts for the ethanol oxidation reaction. The hydrothermal route herein utilized is simple and straightforward, and enables the obtainment of pure nanomaterials with tailored features at moderate reaction times. A comprehensive multi-technique material characterization confirmed the formation of NiO-containing nanoarchitectures, featuring surface hydroxylation along with a tuneable Ni^3+^ content. The combined effect of an open morphology and Ni^3+^ occurrence, regulating the electronic structure and improving catalytic durability, yielded highly attractive EOR performances, the best ones corresponding to a Tafel slope as low as 48 mV/dec and a current density of 150 mA/cm^2^ at 1.6 V vs. RHE. Chemical analysis carried out on the working solutions as a function of reaction time confirmed ethanol conversion into acetate, along with the formation of additional products in lower amounts.

Altogether, the present findings provide a viable strategy for designing non-noble-metal electrocatalysts converting ethanol into added-value products, with interesting implications for a selective electrooxidation of various alcohols in sustainable energy technologies. Future work should focus on decoupling the chemical and electrochemical roles of NiOOH, considering that the regeneration of this species directly governs catalytic activity. In addition, promising research directions encompass additional theoretical and experimental analyses aimed at clarifying the reaction mechanism and optimizing the product selectivity.

## Data Availability

Data supporting this study are available within the article.

## Supplementary Materials

The following supporting information can be downloaded at: https://www.mdpi.com/article/10.3390/nano16080496/s1: Figure S1: XRD patterns for samples synthesized using different HT growth times; Figure S2: Comparison of the Raman signals centred at 500 cm^−1^ for a specimen obtained with a growth time of 60 min and the bare NF substrate; fitting of the peak for the NF substrate, with corresponding band assignments; Figure S3: C1s photopeaks for the target specimens; Table S1: Binding energies and relative contribution of the various components to the overall C1s peak for the target specimens; Figure S4: Ni2p photopeaks for samples obtained for deposition times of 15 min and 60 min; Table S2: Binding energies and relative contribution of the various components to the Ni2p_3/2_ peak for the target samples; Figure S5: O1s photopeaks for samples obtained for deposition times of 15 min and 60 min; Table S3: Binding energies and relative contribution of the various components to the overall O1s peak for the target specimens; Table S4: Energy gap, work function, valence band edge separation from the Fermi level, ionization potential and electron affinity, estimated by REELS and UPS measurements. Figure S6: Electrochromic effect due to the surface oxidation of the sample synthesized using an HT growth time of 30 min to black-colored NiOOH upon performing an LSV anodic scan at *pH* = 14; LSV curves registered in 1.0 M KOH for samples obtained with different deposition times; Figure S7: CV scans pertaining to the electrocatalyst deposited for 30 min upon addition of small EtOH volumes (25, 50, 75 μL) in 1.0 M KOH electrolyte solution; Table S5: Comparison of EOR performances of the actual materials with representative data reported for other Ni-based electrocatalysts. Figure S8: LSV curves recorded in 1.0 M KOH + 1.3 M EtOH solutions on the pristine samples and every 90 days for 6 months upon specimen storage under ambient conditions; Figure S9: Representative FE-SEM images of electrocatalysts obtained at different deposition times after CA testing at 1.6 V Vs. RHE for 24 h; Figure S10: O1s and Ni2p photoelectron peaks for the 15 min and 60 min specimens after completion of the tests indicated in Figure S9; Figure S11: 1D ^13^C-NMR spectrum of the starting solution of ethanol in a KOH aqueous solution; Figure S12: 1D ^13^C-NMR spectrum of the reaction solution after CA testing at 1.6 V Vs. RHE for 3 h. Inset: ^13^C multiplicity without ^1^H decoupling confirms assignment to the methyl group of acetic acid; Figure S13: 1D ^13^C-NMR spectrum of the reaction solution after CA testing at 1.6 V Vs. RHE for 6 h. Inset: ^13^C multiplicity, obtained from NMR experiments without ^1^H decoupling, confirms assignment to glycolate (triplet for -CH_2_- at about 46 ppm and singlet for COO^−^ at about 180 ppm). Figure S14: 1D ^13^C-NMR spectrum of the reaction solution after CA testing at 1.6 V Vs. RHE for 24 h. Inset: ^13^C multiplicity for the very weak peak at about 171 ppm, obtained from NMR experiments without ^1^H decoupling, seeming to point to a doublet, consistent with the presence of a small glycoxylate amount.
