# Supplementary material for: Tailoring NiO-Based Nanostructures for the Electrochemical Valorization of Ethanol: Structure–Property Insights
Source: Nanomaterials (Basel). 2026 Apr 21;16(8):496. doi: 10.3390/nano16080496 (PMC13118891; doi:10.3390/nano16080496)
Supplement: Supplementary file 1 [file nanomaterials-16-00496-s001.zip › nanomaterials-4261327-supplementary.pdf]

Supporting Material

Tailoring NiO-Based Nanostructures for the Electrochemical Valorization of Ethanol: Structure–Property Insights

Ivan Blagojevic [1], Chiara Maccato [1*], Marta De Zotti [1], Davide Barreca [2], Alberto Gasparotto [1], Raffaella Signorini [1], and Gian Andrea Rizzi [1]

[1] Department of Chemical Sciences, Padova University and INSTM, 35131 Padova, Italy; ivan.blagojevic@studenti.unipd.it (I.B.); marta.dezotti@unipd.it (M.D.Z.); alberto.gasparotto@unipd.it (A.G.); raffaella.signorini@unipd.it (R.S.); gianandrea.rizzi@unipd.it (G.A.R.)

[2] CNR-ICMATE and INSTM, Department of Chemical Sciences, Padova University, 35131 Padova, Italy; davide.barreca@cnr.it (D.B.)

* Correspondence: chiara.maccato@unipd.it (C.M.); Tel.: +39-0498275234 (C.M.).

Table of Contents

	Page
**§ S1. Physico-chemical characterization**	S3
**§ S2. Electrochemical functional tests**	S9
**§ S3. Characterization after electrochemical tests**	S14
**§ S4. Nuclear magnetic resonance (NMR) analysis**	S16

§ S1. Physico-chemical characterization

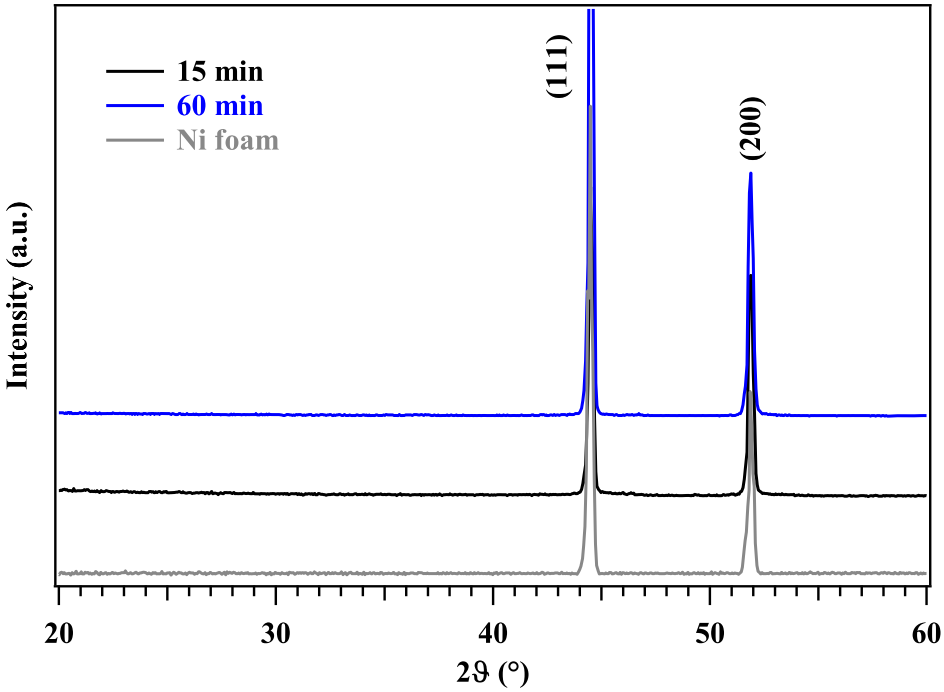

Figure S1. X-ray diffraction (XRD) patterns for samples synthesized using different HT growth times times. The pattern of the bare Ni foam substrate (grey curve) is also plotted for comparison. In all cases, only (111) and (200) reflections from metallic nickel from the substrate [1] could be detected.

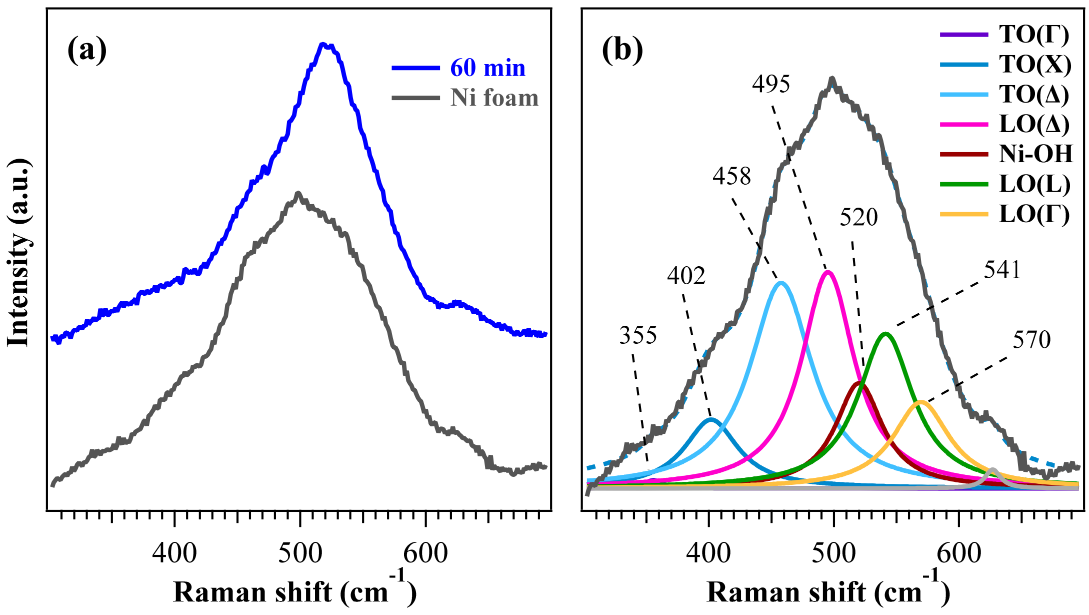

Figure S2. (a) Comparison of the Raman signals centred at 500 cm^{-1} for a specimen obtained with a growth time of 60 min and the bare Ni foam (NF) substrate. (b) Fitting of the peak for the NF substrate, with corresponding band assignments.

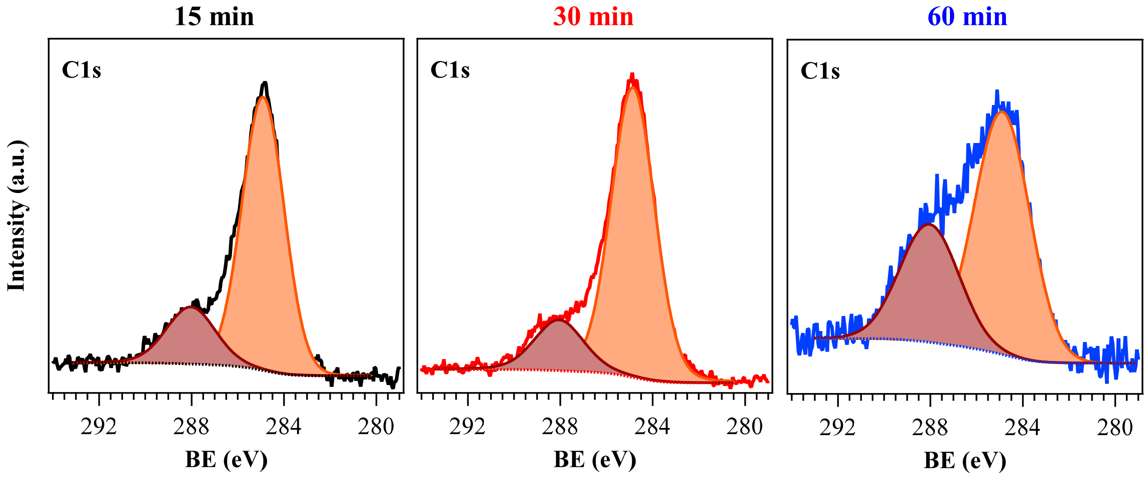

Figure S3. C1s photopeaks for the target specimens.

	C1s	BE (eV)	Area %
15 min	Adventitious C	284.8	79.8
	Chemisorbed carbonates	288.0	20.2
30 min	Adventitious C	284.8	83.0
	Chemisorbed carbonates	288.0	17.0
60 min	Adventitious C	284.8	65.6
	Chemisorbed carbonates	288.0	34.4

Table S1. Binding energies (BEs, eV) and relative contribution (%) of the various components to the overall C1s peak for the target specimens (color codes as in Figure S2). The attribution was made based on previous studies [2-5].

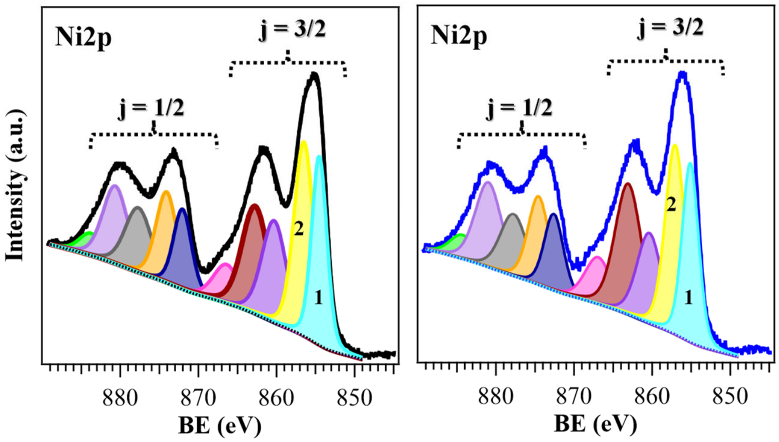

Figure S4. Ni2p photopeaks for samples obtained for deposition times of 15 min (left) and 60 min (right). The two low BE components (labeled as **1** and **2**) are associated respectively to Ni^{2+} and Ni^{3+}.

	$Ni2p_{3/2}$	BE (eV)	Area %
**15 min**	**1** $\propto$ **[Ni^{2+}]**	854.5	22
	**2** $\propto$ **[Ni^{3+}]**	856.5	29
	Satellites	860.3	19
		862.7	22
		866.4	8
**30 min**	**1** $\propto$ **[Ni^{2+}]**	854.4	25
	**2** $\propto$ **[Ni^{3+}]**	856.3	35
	Satellites	860.4	18
		862.5	15
		865.4	7
**60 min**	**1** $\propto$ **[Ni^{2+}]**	855.1	21
	**2** $\propto$ **[Ni^{3+}]**	857.0	27
	Satellites	860.4	18
		863.1	25
		866.9	9

Table S2. BEs (eV) and relative contribution (%) of the various components to the $Ni2p_{3/2}$ peak for the target samples (color codes as in Figures 3b and S4).

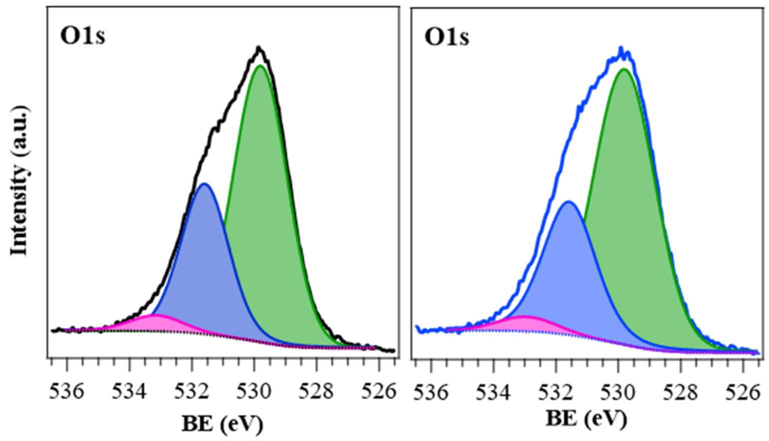

Figure S5. O1s photopeaks for samples obtained for deposition times of 15 min (left panel) and 60 min (right panel).

	O1s	BE (eV)	Area %
**15 min**	Ni-O	529.8	63.4
	Ni-OH	531.5	32.8
	Adsorbed H_2O	533.2	3.8
**30 min**	Ni-O	529.8	46.4
	Ni-OH	531.5	45.8
	Adsorbed H_2O	533.4	7.8
**60 min**	Ni-O	529.8	66.3
	Ni-OH	531.6	30.4
	Adsorbed H_2O	533.0	3.2

Table S3. BEs (eV) and relative contribution (%) of the various components to the overall O1s peak for the target specimens (color codes as in Figures 3c and S4). The attribution was made based on previous studies [5-9].

	15 min	30 min	60 min
**E_G (eV)**	2.9	2.6	2.7
**Φ (eV)**	4.9	4.7	5.0
**E_F - VB (eV)**	1.9	1.8	2.0
**IP (eV)**	5.9	5.5	5.7
**EA (eV)**	4.0	3.9	4.3

Table S4. Energy gap (E_G), work function [Φ, difference between vacuum level energies and Fermi energies (E_F)], valence band edge separation from the Fermi level (E_F – VB), ionization potential (IP) and electron affinity (EA), estimated by reflection electron energy loss spectroscopy (REELS) and ultraviolet photoelectron spectroscopy (UPS) measurements. IP (EA) are evaluated as the energy difference between valence (conduction) band edges (VB and CB, respectively) and the vacuum level energy [3,5].

§ S2. Electrochemical functional tests

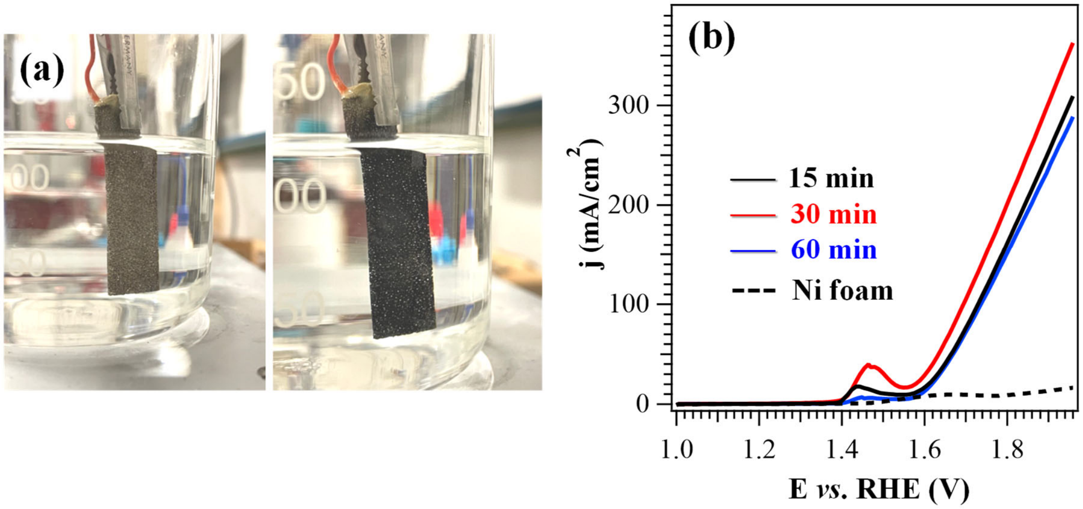

Figure S6. (a) Electrochromic effect due to the surface oxidation of the sample synthesized using an HT growth time of 30 min (left picture) to black-colored NiOOH (right picture) upon performing a linear sweep voltammetry (LSV) anodic scan at $pH = 14$. (b) LSV curves registered in 1.0 M KOH for samples obtained with different deposition times. The potentials required to reach current densities of 100 mA/cm^2 were 1.74 V, 1.70 V, and 1.73 V *vs*. RHE for the 15 min, 30 min, and 60 min specimens, respectively. The curve for the bare Ni foam substrate, which delivered a significantly lower current density than the target specimens, is also reported for comparison.

For the 30 min sample, its peculiar morphology combined with the presence of NiOOH and a defect-rich NiO phase may generate or promote highly active sites for oxygen evolution. This behavior is driven by Ni^{3+} centers, which are well recognized as a key factor in water electrooxidation. In this regard, the higher abundance of Ni(III) can be deemed to be favorable for the formation of OER intermediates, ultimately accounting for the observed enhancement in OER activity [10].

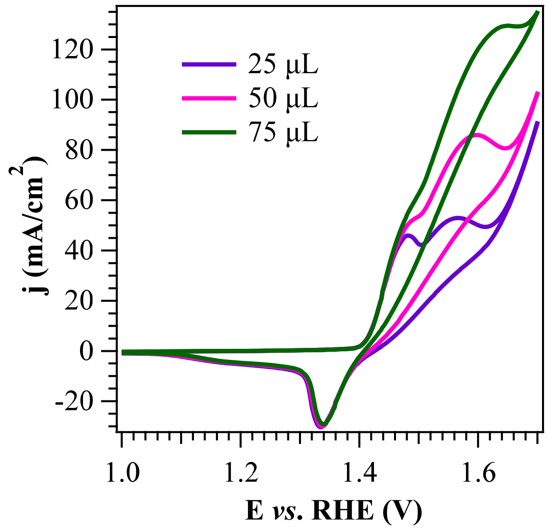

Figure S7. Cyclic voltammetry (CV) scans pertaining to the electrocatalyst deposited for 30 min upon addition of small EtOH volumes (25, 50, 75 μL) in 1.0 M KOH electrolyte solution.

.

Catalyst	Electrolyte	j @ 1.60 V *vs.* RHE (mA/cm^2)	$E_{j=10}$ (V)	Tafel slope (mV/dec)	Ref.
NiO HT 15′	1 M KOH + 1.3 M EtOH	100	1.44	52	This study
NiO HT 30′		150	1.41	48	
NiO HT 60′		74	1.43	61	
NiO	1.0 M KOH + 1.0 M EtOH	> 60	0.7	102	[11]
NiO		≈6.2	1.66	158	[12]
NiO		69.4	≈1.36	325	[13]
NiO_x		> 90	1.40	46.6	[14]
NiO_x/CN_x		> 90	1.35	30.2	[14]
NiO	1.0 M KOH + 0.5 M EtOH	≈100	1.12	468.2	[6]
NiO	1.0 M KOH + 0.05 M EtOH	12.5	1.56	*n.a.*	[15]
Ni_2O_3	1.0 M KOH + 1.0 M EtOH	≈70	*n.a.*	62	[16]
$Ni(OH)_2$ NSs		≈48.0	1.43	*n.a.*	[17]
$Ni(OH)_2$ MSs		≈21.0	1.47	*n.a.*	[18]
β-$Ni(OH)_2$		≈32	1.40	44	[19]
$Ni(OH)_2$ NSs		> 200	1.31÷1.32	25.3÷46.9	[20]
$Ni(OH)_2$		≈172	≈1.38	*n.a.*	[21]
β-$Ni(OH)_2$	1.0 M KOH + 0.5 M EtOH	≈11	1.50	*n.a.*	[22]
NiOOH	1.0 M KOH + 1.0 M EtOH	≈125	1.37	53	[23]
NiOOH		≈250	≈1.34	69.1	[24]
Cu-doped NiOOH		≈190	1.35	24	[23]
NiOOH	1.0 M KOH + 1.5 M EtOH	≈200 **	1.37	91	[25]
NiO@CeO_2-1:1	1.0 M KOH + 1.0 M EtOH	≈30	1.46	50	[12]
$Ni_{0.67}Co_{0.33}O_x$		126	≈1.34	187.9	[13]
$Ni_{0.33}Co_{0.67}O_x$		82	≈1.36	258.4	[13]

Catalyst	Electrolyte	j @ 1.60 V *vs.* RHE (mA/cm^2)	$E_{j=10}$ (V)	Tafel slope (mV/dec)	Ref.
Ni_3S_2 NWs	1.0 M KOH + 1.0 M EtOH	≈162	1.35	*n.a.*	[26]
NiS-NiS_2/CC *	1.0 M KOH + 1.0 M EtOH	≈150	1.39	*n.a.*	[27]
NiS	1.0 M KOH + 1.0 M EtOH	≈130	1.34	*n.a.*	[28]
NiS		> 150	1.37	40.1	[29]

Table S5. Comparison of EOR performances of the actual materials with representative data reported for other Ni-based electrocatalysts. $E_{j=10}$ indicates the bias value necessary to reach a current density of 10 mA/cm^2. *n.a.* = data not available; NSs = nanosheets; MSs = microspheres; NWs = nanowires; NRs = nanorods; * = nickel sulphide heterostructure embedded in biomass carbon.

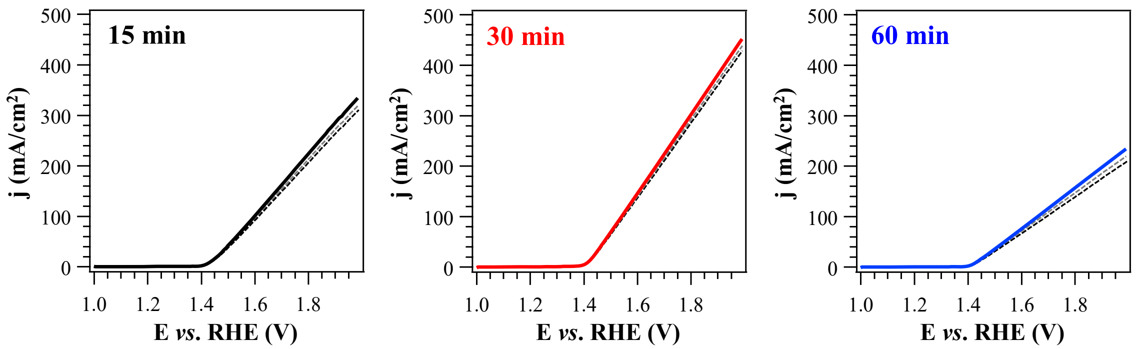

Figure S8. LSV) curves recorded in 1.0 M KOH + 1.3 M EtOH solutions on the pristine samples (solid lines) and every 90 days for 6 months upon specimen storage under ambient conditions (dashed lines).

§ S3. Characterization after electrochemical tests

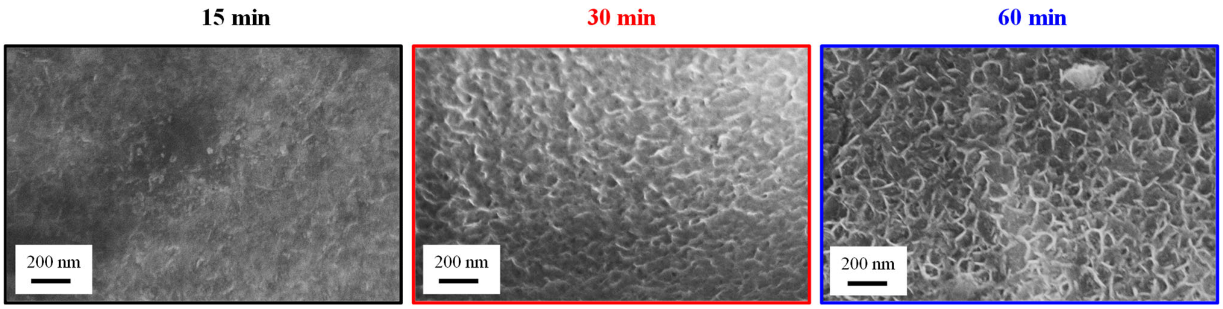

Figure S9. Representative FE-SEM images of electrocatalysts obtained at different deposition times after CA testing at 1.6 V *vs*. RHE for 24 h.

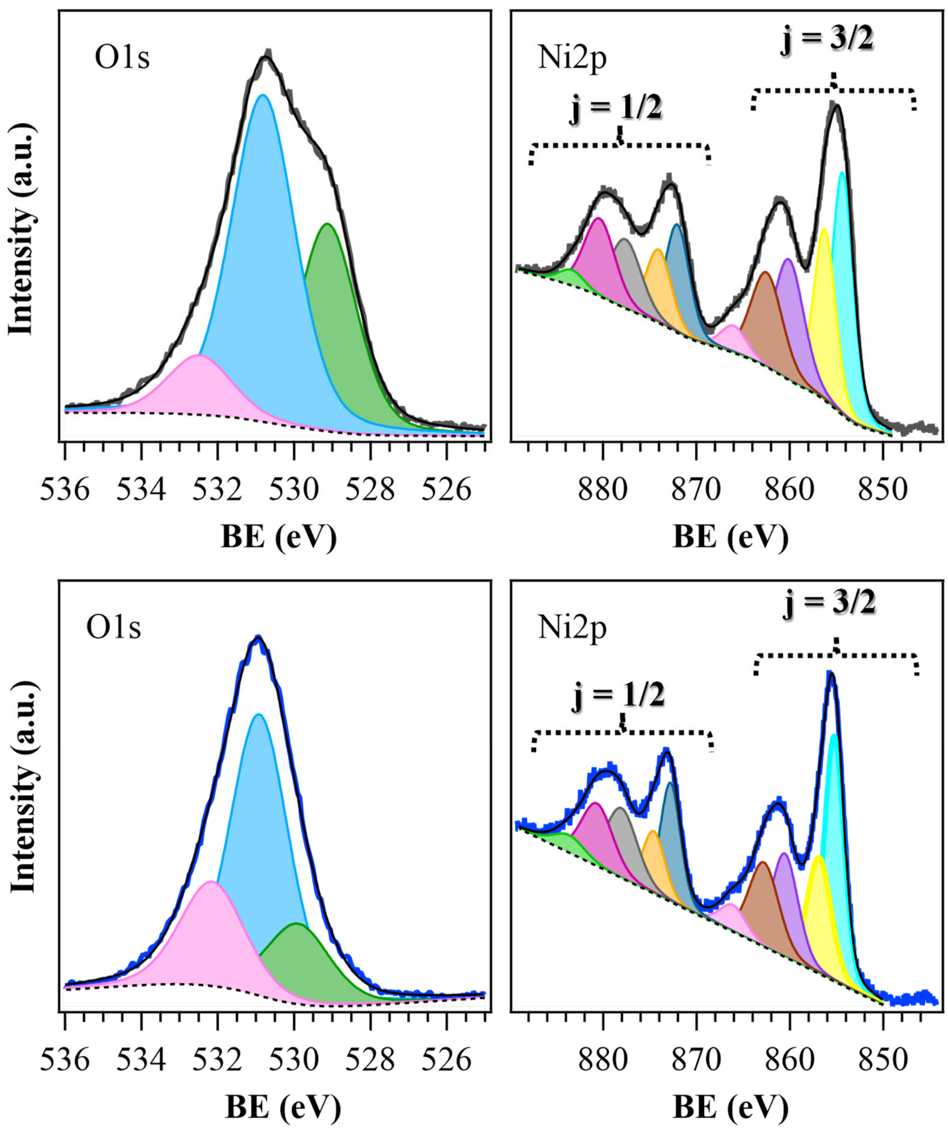

Figure S10. O1s and Ni2p photoelectron peaks for the 15 min (top panel, black) and 60 min (bottom panel, blue) specimens after completion of the tests indicated in Figure S9. Color codes as in Figures S4 and S5.

§ S4. Nuclear magnetic resonance (NMR) analysis

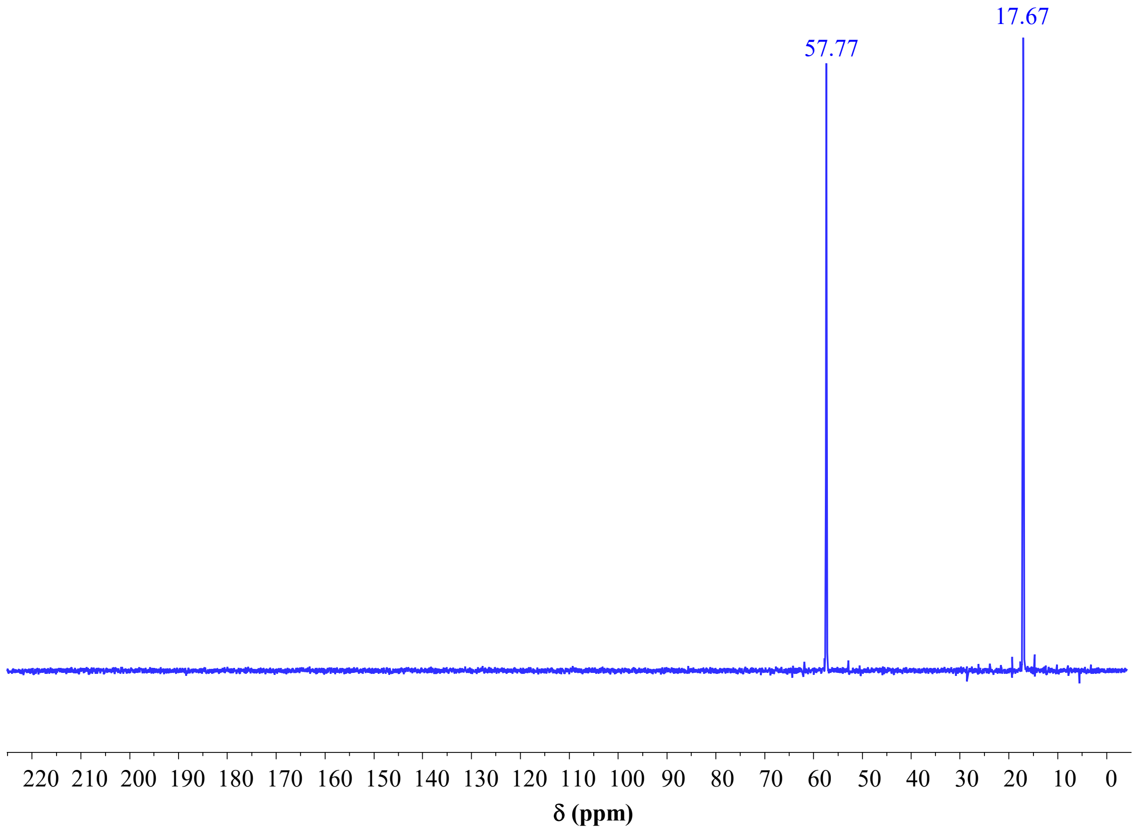

Figure S11. 1D ^{13}C-NMR spectrum of the starting solution of ethanol in a KOH aqueous solution (H_2O/D_2O 9:1, 100.6 MHz, 298 K).

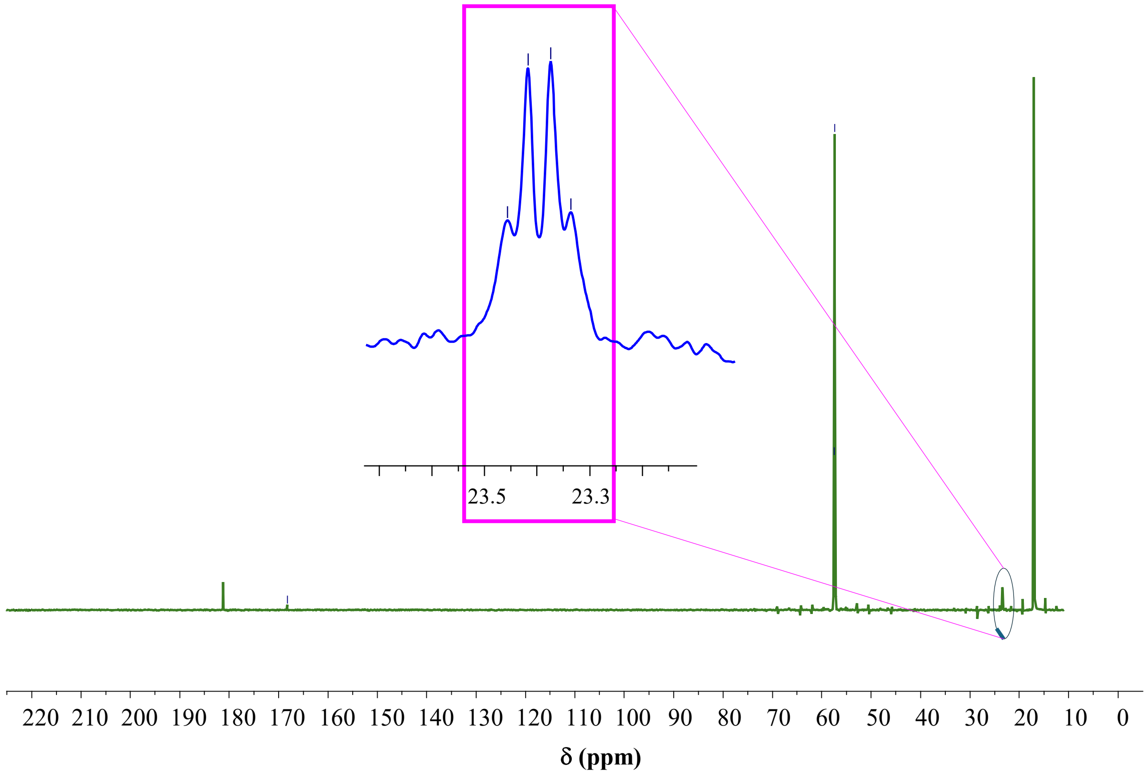

Figure S12. 1D ^{13}C-NMR spectrum of the reaction solution after chronoamperometry (CA) testing at 1.6 V *vs*. the reversible hydrogen electrode (RHE) for 3 h (H_2O/D_2O 9:1, 100.6 MHz, 298K). Inset: ^{13}C multiplicity without ^{1}H decoupling confirms assignment to the methyl group of acetic acid.

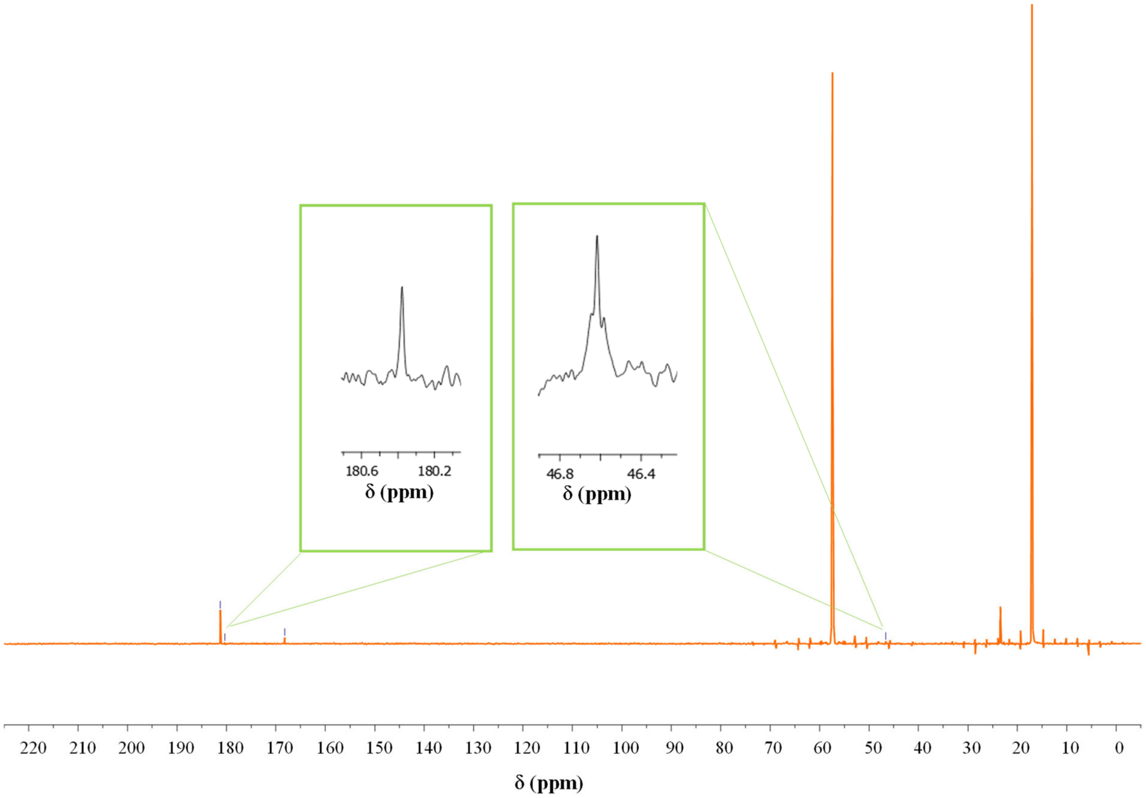

Figure S13. 1D ^{13}C-NMR spectrum of the reaction solution after CA testing at 1.6 V *vs*. RHE for 6 h (H_2O/D_2O 9:1, 100.6 MHz, 298 K). Inset: ^{13}C multiplicity, obtained from NMR experiments without ^{1}H decoupling, confirms assignment to glycolate (triplet for -CH_2- at about 46 ppm and singlet for COO^- at about 180 ppm).

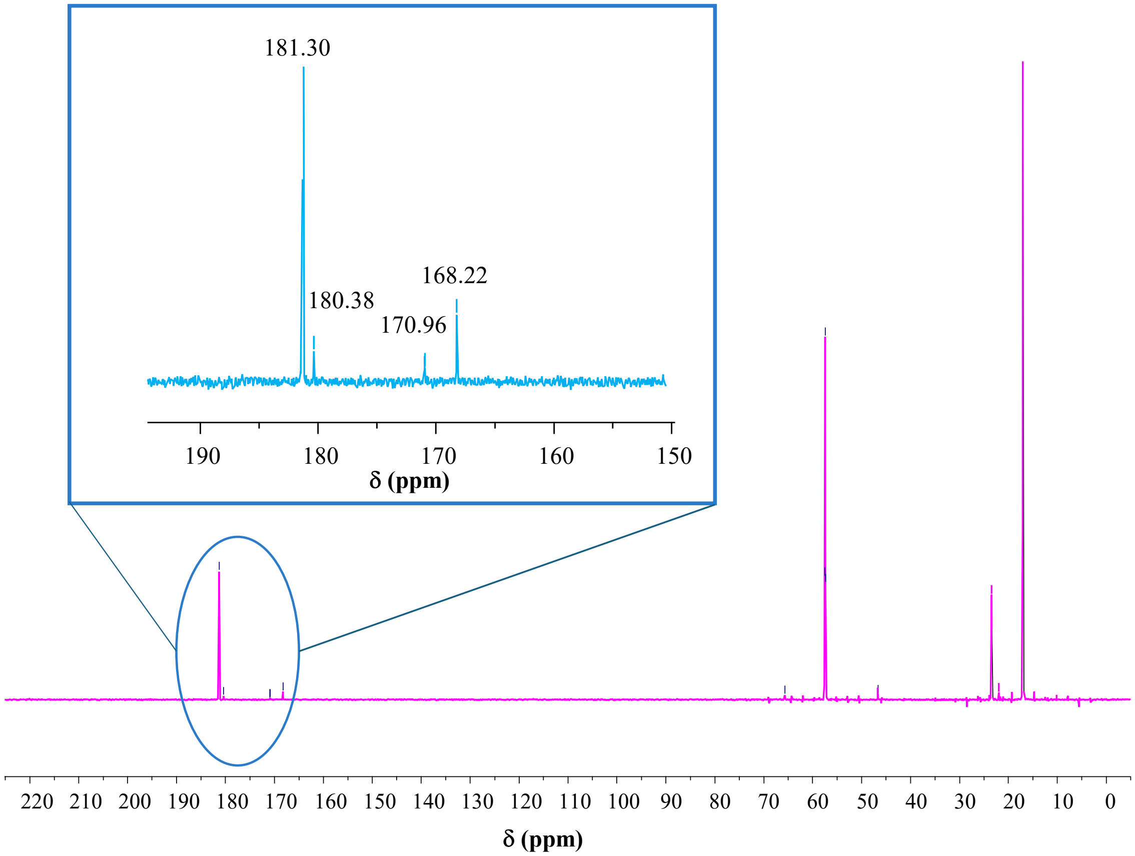

Figure S14. 1D ^{13}C-NMR spectrum of the reaction solution after CA testing at 1.6 V *vs*. RHE for 24 h (H_2O/D_2O 9:1, 100.6 MHz, 298 K). Inset: ^{13}C multiplicity for the very weak peak at about 171 ppm, obtained from NMR experiments without ^{1}H decoupling, seeming to point to a doublet, consistent with the presence of a small glycoxylate amount.

References

1. PDF card no. 04-0850 (2002).
2. Pagot, G.; Benedet, M.; Maccato, C.; Barreca, D.; Di Noto, V. XPS study of NiO thin films obtained by chemical vapor deposition. *Surf. Sci. Spectra* **2023**, *30*, 024028, doi:https://doi.org/10.1116/6.0003008.
3. Maccato, C.; Barreca, D.; Signorin, L.; Scattolin, E.; Tabacchi, G.; Fois, E.; Sada, C.; Lebedev, O.I.; Gasparotto, A.; Modin, E., et al. Plasma-assisted fabrication of NiO nanoarchitectures: from design to oxygen evolution electrocatalysis. *Catal. Sci. Technol.* **2025**, *15*, 6358-6371, doi:https://doi.org/10.1039/D5CY00833F.
4. Barreca, D.; Maccato, C.; Gasparotto, A.; Rizzi, G.A. XPS analysis of F-containing NiO nanoarchitectures fabricated by plasma-assisted chemical vapor deposition. *Surf. Sci. Spectra* **2025**, *32*, 024005, doi:https://doi.org/10.1116/6.0004637.
5. Benedet, M.; Maccato, C.; Pagot, G.; Invernizzi, C.; Sada, C.; Di Noto, V.; Rizzi, G.A.; Fois, E.; Tabacchi, G.; Barreca, D. Growth of NiO thin films in the presence of water vapor: insights from experiments and theory. *J. Phys. Chem. C* **2023**, *127*, 22304-22314, doi:https://doi.org/10.1021/acs.jpcc.3c05067.
6. Luo, T.; Xie, J.; Li, X.; Wang, X.; Sun, L.; Bu, F.; Zhao, B.; Kan, W.; He, Y. Electronic structure engineering of ultradispersed Ru in NiO nanobricks for selective and efficient ethanol electrooxidation. *J. Colloid Interface Sci.* **2026**, *708*, 139791, doi:https://doi.org/10.1016/j.jcis.2025.139791.
7. Barreca, D.; Scattolin, E.; Maccato, C.; Gasparotto, A.; Signorin, L.; El Habra, N.; Šuligoj, A.; Štangar, U.L.; Rizzi, G.A. Controllable properties of NiO nanostructures fabricated by plasma assisted-chemical vapor deposition. *Chem. Commun.* **2025**, *61*, 2945-2948, doi:https://doi.org/10.1039/d4cc06548d.
8. Scattolin, E.; Benedet, M.; Rizzi, G.A.; Gasparotto, A.; Lebedev, O.I.; Barreca, D.; Maccato, C. Graphitic carbon nitride structures on carbon cloth containing ultra- and nano-dispersed NiO for photoactivated oxygen evolution. *ChemSusChem* **2024**, *17*, e202400948, doi:https://doi.org/10.1002/cssc.202400948.
9. Zhao, M.; Yang, X.; Fu, Z.; Wang, W.; Wen, W.; Xiao, H.; Zhang, L.; Zhang, J.; Lv, B.; Jia, J. Facile electrolysis-solvothermal synthesis of NiO_x/graphene for enhanced ethanol oxidation to acetate. *Dalton Trans.* **2024**, *53*, 4237-4242, doi:https://doi.org/10.1039/D3DT03963C.
10. Steimecke, M.; Seiffarth, G.; Schneemann, C.; Oehler, F.; Förster, S.; Bron, M. Higher-valent nickel oxides with improved oxygen evolution activity and stability in alkaline media prepared by high-temperature treatment of $Ni(OH)_2$. *ACS Catal.* **2020**, *10*, 3595-3603, doi:https://doi.org/10.1021/acscatal.9b04788.
11. Sood, K.; Arora, A.; Bhasin, A.K.K.; Bhasin, K.K.; Jha, M. Green synthesis of NiO nanoparticles using waste tea extract and their utilization as potential electroanode for ethanol oxidation. *Energy Technol.* **2025**, *13*, 2500280, doi:https://doi.org/10.1002/ente.202500280.
12. Zhu, X.; Wang, N.; Xu, W.; Zhou, W.; An, M.; Zhao, D.; Zhou, Y.; Li, L. NiO coupled with CeO_2 as an efficient electrocatalysts for ethanol oxidation in hybrid water splitting. *Int. J. Hydrogen Energy* **2024**, *51*, 1451-1455, doi:https://doi.org/10.1016/j.ijhydene.2023.11.133.
13. Zhang, Y.; Liu, R.; Ma, Y.; Jian, N.; Pan, H.; Liu, Y.; Deng, J.; Li, L.; Shao, Q.; Li, C., et al. Nickel–cobalt oxide nanoparticles as superior electrocatalysts for enhanced coupling hydrogen evolution and selective ethanol oxidation reaction. *J. Mater. Chem. A* **2024**, *12*, 17252-17259, doi:https://doi.org/10.1039/D4TA03259D.
14. Shekhawat, A.; Samanta, R.; Panigrahy, S.; Barman, S. Electrocatalytic oxidation of urea and ethanol on two-dimensional amorphous nickel oxide encapsulated on N-doped carbon nanosheets. *ACS Appl. Energy Mater.* **2023**, *6*, 3135-3146, doi:https://doi.org/10.1021/acsaem.3c00151.

15. Chen, W.; Shi, J.; Xie, C.; Zhou, W.; Xu, L.; Li, Y.; Wu, Y.; Wu, B.; Huang, Y.-C.; Zhou, B., et al. Unraveling the electrophilic oxygen-mediated mechanism for alcohol electrooxidation on NiO. *Natl. Sci. Rev.* **2023**, *10*, nwad099, doi:https://doi.org/10.1093/nsr/nwad099.
16. Du, R.; Zhong, Q.; Tan, X.; Liao, L.; Tang, Z.; Chen, S.; Yan, D.; Zhao, X.; Zeng, F. Optimized Electrodeposition of Ni_2O_3 on Carbon Paper for Enhanced Electrocatalytic Oxidation of Ethanol. *ACS Omega* **2024**, *9*, 30404-30414, doi:https://doi.org/10.1021/acsomega.4c01658.
17. Zhang, Z.; Dong, Y.; Carlos, C.; Wang, X. Surface ligand modification on ultrathin $Ni(OH)_2$ nanosheets enabling enhanced alkaline ethanol oxidation kinetics. *ACS Nano* **2023**, *17*, 17180-17189, doi:https://doi.org.10.1021/acsnano.3c05014.
18. Lidasan, J.J.B.; del Rosario, J.A.D.; Ocon, J.D. Ethanol electrooxidation on phase- and morphology-controlled $Ni(OH)_2$ microspheres. *Catalysts* **2020**, *10*, 740, doi:https://doi.org/10.3390/catal10070740.
19. Kumari, S.; Sunaina; Devi, S.; Jha, M. Excellent ethanol oxidation and oxygen evolution reaction from ultrafine nickel hydroxide nanorods stabilized at room temperature. *Mater. Sci. Eng., B* **2024**, *306*, 117445, doi:https://doi.org/10.1016/j.mseb.2024.117445.
20. Ma, S.; Chen, D.; Ye, Z.; Wang, Y.; Xu, J.; Zhang, J. Constructing $Ni(OH)_2$ nanosheets on a nickel foam electrode for efficient electrocatalytic ethanol oxidation. *Dalton Trans.* **2025**, *54*, 14349-14358, doi:https://doi.org/10.1039/D5DT01777G.
21. Zhang, Y.; Xu, R.; Shao, Q.; Luo, G.; Liu, J.; Chen, X.; Yu, S.-s.; Liu, S.; Zhang, J.; Liu, D., et al. Synergistically enhanced $Ni_3Co_1(OH)_x$@Ni_3S_2 heterojunction catalyst for high-performance ethanol electrooxidation. *ACS Appl. Mater. Interfaces* **2025**, *17*, 52371-52381, doi:https://doi.org/10.1021/acsami.5c13244.
22. Chen, W.; Xie, C.; Wang, Y.; Zou, Y.; Dong, C.-L.; Huang, Y.-C.; Xiao, Z.; Wei, Z.; Du, S.; Chen, C., et al. Activity Origins and Design Principles of Nickel-Based Catalysts for Nucleophile Electrooxidation. *Chem* **2020**, *6*, 2974-2993, doi:https://doi.org/10.1016/j.chempr.2020.07.022.
23. Wang, H.; Guan, A.; Zhang, J.; Mi, Y.; Li, S.; Yuan, T.; Jing, C.; Zhang, L.; Zhang, L.; Zheng, G. Copper-doped nickel oxyhydroxide for efficient electrocatalytic ethanol oxidation. *Chin. J. Catal.* **2022**, *43*, 1478-1484, doi:https://doi.org/10.1016/S1872-2067(21)63995-5.
24. Sun, H.; Li, L.; Chen, Y.; Kim, H.; Xu, X.; Guan, D.; Hu, Z.; Zhang, L.; Shao, Z.; Jung, W. Boosting ethanol oxidation by NiOOH-CuO nano-heterostructure for energy-saving hydrogen production and biomass upgrading. *Appl. Catal., B* **2023**, *325*, 122388, doi:https://doi.org/10.1016/j.apcatb.2023.122388.
25. Ni, M.; Tan, M.; Luo, K.; Jiang, D.; Yuan, Y.; Zhu, C.; Du, H.; Ren, H. Electro-oxidation of alcohols over electrochemically activated nickel alloys for energy-saving hydrogen production. *J. Mater. Chem. A* **2024**, *12*, 22550-22556, doi:https://doi.org/10.1039/D4TA02707H.
26. Zhang, Y.; Zhu, W.; Fang, J.; Xu, Z.; Xue, Y.; Liu, D.; Sui, R.; Lv, Q.; Liu, X.; Wang, Y., et al. Defective Ni_3S_2 nanowires as highly active electrocatalysts for ethanol oxidative upgrading. *Nano Res.* **2022**, *15*, 2987-2993, doi:https://doi.org/10.1007/s12274-021-3930-x.
27. Zhao, Q.; Wang, L.; Fang, L.; Chen, W.; Zhuang, Z.; Gan, R.; Ma, Q.; Ran, Y.; Zhang, Y.; Wang, D. Sulfur form regulation and dual-interface effect: catalytic mechanism of nickel-based catalyst in ethanol electrooxidation. *J. Colloid Interface Sci.* **2026**, *701*, 138689, doi:https://doi.org/10.1016/j.jcis.2025.138689.
28. Li, J.; Tian, X.; Wang, X.; Zhang, T.; Spadaro, M.C.; Arbiol, J.; Li, L.; Zuo, Y.; Cabot, A. Electrochemical conversion of alcohols into acidic commodities on nickel sulfide nanoparticles. *Inorg. Chem.* **2022**, *61*, 13433-13441, doi:https://doi.org/10.1021/acs.inorgchem.2c01695.
29. Wang, Z.; Liao, X.; Zhou, M.; Huang, F.; Owusu, K.A.; Li, J.; Lin, Z.; Sun, Q.; Hong, X.; Sun, C., et al. Interfacial and vacancies engineering of copper nickel sulfide for enhanced oxygen reduction and alcohols oxidation activity. *Energy Environ. Mater.* **2025**, *6*, e12409, doi:https://doi.org/10.1002/eem2.12409.
